# Adipocyte Hypertrophy, Fatty Liver and Metabolic Risk Factors in South Asians: The Molecular Study of Health and Risk in Ethnic Groups (mol-SHARE)

**DOI:** 10.1371/journal.pone.0022112

**Published:** 2011-07-28

**Authors:** Sonia S. Anand, Mark A. Tarnopolsky, Shirya Rashid, Karleen M. Schulze, Dipika Desai, Andrew Mente, Sandy Rao, Salim Yusuf, Hertzel C. Gerstein, Arya M. Sharma

**Affiliations:** 1 Population Health Research Institute, Hamilton Health Sciences and McMaster University, Hamilton, Ontario, Canada; 2 Department of Medicine, McMaster University, Hamilton, Ontario, Canada; 3 Department of Pediatrics, McMaster University, Hamilton, Ontario, Canada; 4 Department of Clinical Epidemiology/Biostatistics, McMaster University, Hamilton, Ontario, Canada; 5 University of Alberta, Edmonton, Alberta, Canada; Hospital for Sick Children, Canada

## Abstract

**Objective:**

We sought to determine if differences in the distribution and characteristics of adipose tissue between South Asians and white Caucasians account for differences in risk factors for cardiovascular disease.

**Research Design and Methods:**

We recruited 108 healthy South Asians (36.8 years) and white Caucasians (34.2 years) within three BMI strata. Body composition, adipocyte size, abdominal fat area, and hepatic adiposity were assessed and related to fasting glucose, insulin, lipids and adiponectin.

**Results:**

After adjustment for age, sex, and BMI, South Asians compared to white Caucasians had higher ln fasting insulin (mean difference (md): 0.44; 95% CI: 0.20–0.69), lower HDL cholesterol (md: −0.13; 95% CI:−0.26 to −0.01), and lower adiponectin (md: −2.38; 95% CI: −3.59 to −1.17). South Asians also had more body fat (md: 2.69; 95% CI: 0.70 to 4.69), lower lean muscle mass (md: −3.25; 95%CI: −5.35 to −1.14), increased waist to hip ratio (md: 0.03; 95% CI: 0.01–0.05), less superficial subcutaneous abdominal adipose tissue (md: −2.94; 95% CI: −5.56 to−0.32), more deep/visceral to superficial adipose tissue ratio (md 0.34; 95% CI: 0.02 to 0.65), and more liver fat (md: 7.43%; 95% CI: 2.30 to 12.55%). Adipocyte area was increased in South Asians compared to white Caucasians (md: 64.26; 95% CI: 24.3 to 104.1) units^2^. Adjustment for adipocyte area attenuated the ethnic differences in insulin (md: 0.22; 95% CI: −0.07 to 0.51), HDL (md: −0.01; 95% CI: −0.16 to 0.13) and adiponectin (md: −1.11; 95% CI: −2.61 to 0.39). Adjustment for differences in adipocyte area and fat distribution attenuated the ethnic difference in liver fat (md: 5.19; 95% CI: 0.31 to 10.06).

**Conclusion:**

South Asians have an increased adipocyte area compared to white Caucasians. This difference accounts for the ethnic differences in insulin, HDL cholesterol, adiponectin, and ectopic fat deposition in the liver.

## Introduction

South Asians (people who originate from the Indian subcontinent) are more likely to develop type 2 diabetes and myocardial infarction (MI) at younger ages compared to white Caucasians of European origin [1]–[3]. Recent evidence suggests that South Asians develop changes in metabolic risk factors for cardiovascular disease (CVD), such as glucose, insulin, lipid levels and adipokines at significantly lower body mass indices than white Caucasians [4]. This may be due to a higher total body fat, and higher ectopic fat deposition in the abdomen, liver and elsewhere. This ectopic fat distribution may occur as a result of a diminished storage capacity of superficial subcutaneous adipose tissue leading to an overflow of fatty acids to ectopic sites where it may affect structure and/or function [5]–[7]. We investigated whether differences in the amount of total fat, its distribution and adipocyte characteristics can account for differences in metabolic risk factors for CVD (i.e. glucose, insulin, lipids, adiponectin) in South Asians compared to white Caucasians.

## Methods

This study was approved by the Hamilton Health Sciences/Faculty of Health Sciences Research Ethics Board on July 25, 2005 and written informed consent was obtained from each participant.

### Recruitment

Men and women of South Asian origin (defined as parents and grandparents who originated from India, Pakistan, Sri Lanka, or Bangladesh) and white Caucasians (ancestors originated from Europe) were consecutively recruited into one of three BMI strata: ≤25 kg/m^2^, 26–29 kg/m^2^, ≥30 kg/m^2^, and matched on sex and age (+/−5 years). Individuals with established cardiovascular disease and/or with previously diagnosed type 2 diabetes were excluded. Participants were recruited by public advertisements in temples, hospitals, on the university campus, and by letters mailed to homes in geographic areas where high concentrations of South Asians lived. Potential participants were assessed for eligibility over the telephone, and if eligible and agreeable to the study protocol, they were invited to complete two consecutive visits.

### Measurements and Methods

#### Biochemical Measurements

Blood samples were taken from all participants after a 12 hour fast. Total serum cholesterol and glucose were measured using enzymatic methods [8], [9]. Serum LDL cholesterol was calculated using the Friedewald formula [10], and HDL cholesterol was measured using a homogenous enzymatic colorimetric assay (ROCHE/Hitachi Modular Package Insert). Triglycerides were measured using the enzymatic colorimetric assay on the ROCHE/Hitachi Modular instrument and reagent kit. Insulin was measured on the Roche Elecsys R 2010 immunoassay analyzer using an electrochemiluminescence immunoassay (Roche Diagnostics GmbH, Indianapolis, Indiana, USA). Serum CRP was measured on the Roche Hitachi 917 using the Tina-quant R CRP high sensitive immunoturbidimetric assay. Analysis of adiponectin was performed using a manual qualitative sandwich immunoassay technique (ELISHA) kit manufactured by R&D Systems Inc (Minneapolis, Minnesota, USA). Basal insulin resistance was calculated using the previously validated homeostatic model assessment index (HOMA-IR) model [11].

#### Body Composition Assessment

Height and weight, and waist and hip circumference were measured using standard methods [2]. Total body fat was measured by Dual-energy X-ray absorptiometry (DXA) after an overnight fast [12]. Detailed descriptions of the methods used to assess body composition, abdominal and liver adipose tissue measurements, and adipose and muscle biopsies are found in the study protocol posted on-line [12].

#### Abdominal Adipose Tissue Measurements

Abdominal visceral and subcutaneous fat area were assessed by magnetic resonance imaging (MRI) performed on a 1.5T whole body MR system (Siemens Symphony). After 3-plane localizer image acquisition, breath-hold axial T1-weighted images at the level of mid-L4 (TR 400 ms, TE 13 ms) were acquired. The volumes of subcutaneous (superficial distinguished from deep subcutaneous tissue where possible) and visceral fat were determined by manual tracing of the areas of fat at the laboratory of Dr. Scott Lear. Intrahepatic fat was assessed using localized single voxel (1–2 cc volume) proton spectra using a phased-array body coil for radiofrequency signal receiving [12].

#### Adipose Tissue Biopsy

Subcutaneous fat biopsies were collected from the superficial subcutaneous fat ∼2–3 cm below skin in the periumbilical region using a Bergstrom needle modified with suction, under local anaesthesia. Fragments of adipose tissue (∼10 mg) were preserved in 40% formalin for subsequent embedding in paraffin, cutting and staining for macrophages with a Mac3 antibody. 50–60 mg of fresh adipose tissue were conserved in 3 mL OsO4 +2.2 mL collidine HCl digested, stained with hemotyxylin and eosin and aliquoted and placed onto a glass slide. From these slides, digital images (20/person) were created.

#### Adipocyte Area Measurement

Adipocyte area and maximum diameter were determined using a software program utilizing the NIH's open-share Image J Software program [13]. The macros were set up to analyze the digested, fixed onto slides, stained (with osmium, hematoxylin and eosin staining) and photographed approximately 50,000 adipocytes from 79 participants. The application was for the photographed slides of the adipocytes. The macros developed determined which cells to include in the calculations of size (as they had to be above a certain level of circularity and size so that no pre-adipocytes or other cell types would be included in the calculations of size), and to calculate the area, width, height, Feret's diameter, minimum Feret's diameter, circularity, perimeter for each adipocyte assigned to each subject.

#### Skeletal Muscle Measurements

Habitual physical activity was assessed with validated physical activity questionnaires [14], and a direct assessment of fitness and strength was made [12]. Subjects were exercised for 15 minutes, the first 5 minutes at a workload of 15 Watts and the next 10 minutes at a workload of 50 Watts. Cardiovascular fitness (VO_2_max) was determined at rest, at 5 minutes into exercise, after 15 minutes of exercise, and 5 minutes after stopping exercise in all subjects using an electronically braked cycle ergometer, and computerized open-circuit gas collection system. Muscle biopsies were taken from the vastus lateralis using a Bergstrom needle modified with suction, under local anaesthesia. Approximately 15 mg of tissue was dissected free and immediately placed into 2% glutaraldehyde for transmission electron microscopy (TEM) to quantify lipid droplet characteristics as previously described [15]. The total number of lipids, mean lipid area, percent lipid area density, and the percentage of lipids touching mitochondria were determined using imaging software (Image Pro Plus, Media Cybernetics, Silver Springs, MD). The method and reliability of the IMCL calculation has been established by the Tarnopolsky lab and published previously [15]. *All biochemical, body fat, adipocyte and muscle analyses were performed by readers who were blinded to participants ethnicity*.

### Statistical

#### Power

We aimed to recruit 60 subjects per ethnic group distributed equally in 3 BMI strata and approximately balanced by age and sex. We estimated this sample size would provide more than 80% power to detect an absolute difference comparing South Asians to white Caucasians in total body fat measured by DXA of 3.7%, and an absolute difference in the visceral to superficial adipose tissue ratio as low as 0.26. We recruited 108 subjects and while our power was maintained for the primary outcomes, our power was lower to detect sex differences, and for multivariate regression modelling [16].

#### Analysis

All analyses were performed using SAS version 9.1.3 (Cary, N.C.). The distribution of all continuous variables was examined, and log transformed to achieve normality if required. Generalized Linear Models (PROC GLM) were used to make comparisons between ethnic groups for metabolic parameters and liver fat tertiles. Adjustments for age, sex, and BMI were made for all ethnic comparisons, and additional covariates were added to a maximum of 5 where indicated. These models provided the mean differences between groups and their respective 95% confidence intervals. We used the LSMEANS option to compute adjusted means and standard errors.

## Results

### Baseline Characteristics

One hundred and eight participants were recruited from Hamilton, Canada between November 10, 2005 and March 15, 2009. The baseline characteristics of both groups are presented in Table 1. Briefly, the average age of participants was 35 years, and approximately half were women. All participants were healthy, and few smoked. Although we attempted to recruit subjects in equal proportions across three BMI strata, the average BMI was slightly higher among white Caucasians compared to South Asians (28.6 vs 26.6 kg/m^2^, P<0.05), chiefly due to difficulties in finding healthy South Asians with high BMI and white Caucasians with low BMI. Due to these differences in body size, we adjusted all ethnic comparisons for age, sex and BMI.

**Table 1:** Baseline characteristics of participants.

	Men *		Women *		Overall †		
	South Asian	European	South Asian	European	South Asian	European	P-value
**Number/group**	32	21	24	31	56	52	
**Age (years)**	36.0±1.7	33.1±2.1	37.9±2.0	34.8±1.8	36.8±1.3	34.2±1.4	
**Current Smoking (#)**	2 (6.3%)	3 (14.3%)	0 (0%)	1 (3.2%)	2 (3.6%)	4 (7.7%)	
**Physical Activity score (1 = low,2 = mod,3 = high)**	1.5±0.1	1.9±0.2	1.3±0.1	1.4±0.1	1.4±0.1	1.6±0.1	
**Height (cm)**	174.2±1.2	177.3±1.5	162.2±1.3	164.2±1.1	167.71±0.86	170.95±0.90	0.01
**Weight (kg)**	83.1±2.2	87.9±2.7	67.8±3.0 §	77.6±2.7 §	77.90±0.84	80.27±0.87	0.06
**Body Mass Index (kg/m^2^)**	27.2±0.7	27.7±0.9	25.7±1.2 §	29.1±1.0 §			
**Waist Circumference (cm)**	96.9±1.9	97.6±2.4	87.3±2.7	90.6±2.3	94.14±0.82	92.00±0.86	0.08
**Hip Circumference (cm)**	105.4±1.6	106.5±2.0	103.8±2.3 §	112.3±2.0 §	106.59±0.77	107.93±0.80	0.24
**Waist-to-Hip Ratio**	0.92±0.01	0.92±0.01	0.84±0.01 §	0.80±0.01 §	0.88±0.01	0.85±0.01	0.007
**Systolic Blood Pressure (mm Hg)**	113.4±1.3	114.0±1.6	110.4±2.1	110.5±1.8	112.36±1.15	111.59±1.19	0.65
**Diastolic Blood Pressure (mm Hg)**	75.8±1.3	75.0±1.6	74.0±1.5	73.5±1.3	75.22±0.96	73.86±1.00	0.34
**Glucose (mmol/L)**	5.11±0.08	5.03±0.10	4.91±0.10	4.85±0.09	5.03±0.06	4.92±0.07	0.24
**Ln Insulin (pmol/L)**	4.23±0.11 ||	3.81±0.13 ||	3.99±0.19	3.98±0.14	4.26±0.09	3.81±0.08	0.0006
**HOMA-IR index**	2.87±0.32	1.94±0.39	2.53±0.96	2.54±0.71	3.07±0.42	2.00±0.40	0.08
**HDL (mmol/L)**	1.24±0.06	1.18±0.07	1.27±0.07 §	1.50±0.06 §	1.24±0.04	1.38±0.04	0.03
**Triglycerides (mmol/L)**	1.57±0.19	1.71±0.23	1.31±0.18	1.11±0.15	1.48±0.13	1.33±0.13	0.44
**Total cholesterol/HDL ratio**	3.96±0.24	4.60±0.30	3.80±0.22	3.30±0.19	3.90±0.17	3.81±0.17	0.69
**Adiponectin (ug/mL)**	4.30±0.44 ||	5.79±0.54 ||	6.77±0.75 §	9.38±0.66 §	5.45±0.41	7.83±0.43	0.0002
**hs-CRP (mg/L)**	1.66±0.33	2.22±0.41	1.53±0.95	3.14±0.70	2.11±0.37	2.31±0.36	0.70

* Means (± SE) are adjusted for age,

† Means (± SE) are adjusted for age, sex, BMI ‡ p<0.05 comparing South Asians to Europeans overall,

§ p<0.05 comparing South Asian women to European women,

|| p<0.05 comparing South Asian men to European men; Physical activity score derived from the SHARE activity index (14).

### Metabolic Risk Factors

South Asians compared to white Caucasians had increased insulin (mean difference (md): 0.44; 95% CI: 0.20–0.69 in ln fasting insulin), lower HDL cholesterol (md: −0.13; 95% CI:−0.26 to −0.01) and lower adiponectin (md: −2.38; 95% CI: −3.59 to −1.17). No differences in fasting glucose, LDL cholesterol, blood pressure, or hs-CRP were observed. (Table 1)

### Adipose Tissue Amount, Distribution, and Cell Analysis

Body fat percentage as determined by DXA scan was higher among South Asians compared to white Caucasians (md: 2.69; 95% CI: 0.70 to 4.69). The waist to hip ratio (md: 0.03; 95% CI: 0.01–0.05) was increased among South Asians compared to white Caucasians. Abdominal fat distribution as determined by MRI indicated that South Asians had less superficial subcutaneous tissue, as a percentage of their total abdominal fat, compared to the white Caucasians (md difference: −2.94; 95% CI: −5.56 to−0.32), and South Asians had relatively more deep subcutaneous and visceral fat compared to superficial subcutaneous fat (md in ratio: 0.34; 95% CI:0.02 to0.65). Fat infiltration of the liver was substantially higher among South Asians compared to white Caucasians (md: 7.43%; 95% CI: 2.30 to 12.55).(Table 2) Further, the area of adipocytes was significantly greater among South Asians compared to white Caucasians (md: 64.2; 95% CI: 24.3 to 104.1), as was the maximum adipocyte diameter (md: 20.68; 95% CI: 7.86–33.5). (Table 2)

**Table 2:** Adipose and lean tissue characteristics of participants.

	Men*		Women*		Overall†	
	South Asian	European	South Asian	European	South Asian	European
**Number/group**	32	21	24	31	56	52
**Total Tissue Mass (kg)**	79.4±1.2	82.9±1.5	68.4±1.0	70.0±1.0	74.1±0.8 ‡	76.6±0.9 ‡
**Percent Body Fat by DXA**	28.6±0.9	27.2±1.2	43.0±0.9 §	38.9±0.9 §	35.4±0.7‡	32.7±0.7‡
**Subcutaneous (SC) Fat Area by MRI (cm^2^)**	238.5±10.7	239.4±14.6	282.8±14.4	282.8±13.4	259.8±8.7	260.0±10.0
**Superficial SC Fat (cm^2^)**	100.5±4.9	107.0±6.0	139.0±10.4	157.9±9.2	118.9±5.4	131.9±5.8
**Deep SC Fat (cm^2^)**	154.8±8.9	145.5±11.0	127.6±9.9	123.9±8.5	142.4±7.3	135.6±7.6
**Visceral Fat Area by MRI (cm^2^)**	153.5±8.8	134.5±12.1	97.3±7.3	95.6±6.8	126.8±6.1	117.5±7.0
**Superficial SC % of Total Fat** ||	25.6±1.0	27.8±1.2	38.6±1.6	42.2±1.4	31.7±0.9 ‡	34.6±0.9 ‡
**Deep SC +Visceral Fat/Superficial SC Fat Ratio**	3.15±0.16	2.68±0.19	1.70±0.13	1.46±0.11	2.47±0.11 ‡	2.14±0.11‡
**Liver Fat (%)**	10.8±2.0	4.7±2.6	9.4±2.9 §	0.2±2.7 §	9.9±1.7 ‡	2.5±1.9 ‡
**Adipocyte Area (x100 units^2^)**	444.9±16.5 ¶	383.8±21.4 ¶	464.3±23.8 §	391.1±18.6 §	451.8±13.5 ‡	387.7±14.1 ‡
**Adipocyte maximum diameter**	256.4±5.2 ¶	237.3±6.8 ¶	264.0±7.7 §	239.2±6.0 §	259.0±4.4 ‡	238.4±4.5 ‡
**Total Fat Free Mass (kg)**	59.0±1.2 ¶	62.8±1.5 ¶	41.0±0.8 §	44.0±0.7 §	50.4±0.7 ‡	53.7±0.8 ‡
**Total Body Lean Mass (kg)**	55.9±1.1 ¶	59.6±1.4 ¶	38.5±0.7 §	41.4±0.7 §	47.6±0.7 ‡	50.8±0.8 ‡
**IMCL Area Density (%)**						
**Total**	0.86±0.08	0.99±0.10	0.81±0.15	1.00±0.10	0.83±0.08	1.01±0.07
**Subsarcolemmal**	6.83±0.67	6.24±0.84	6.77±1.21	6.98±0.81	6.76±0.64	6.74±0.61
**Intramyofibrillar**	0.52±0.06	0.65±0.08	0.52±0.13	0.70±0.08	0.52±0.06	0.68±0.06

* Means (± SE) are adjusted for age and BMI,

† Means (± SE) are adjusted for age, sex and BMI,

|| Total fat area refers to: superficial + deep+visceral fat by MRI,

‡ p<0.05 comparing South Asians to Europeans overall,

§ p<0.05 comparing South Asian women to European women,

¶ p<0.05 comparing South Asian men to European men,

Note: There was no change in the IMCL change after adjustment for peak exercise capacity i.e. Vo2 max at 15 mins.

### Muscle Tissue Characteristics

Using DXA scan, South Asians had significantly lower lean body mass compared to white Caucasians (md: −3.25; 95% CI: −5.35 to −1.14).(Table 2) Skeletal muscle biopsy specimens were analyzed for intramyocellular lipid content (IMCL) and no differences in the area density of total, sub-sarcolemmal and intramyofibrillar IMCL were observed between the groups, even after adjustment for the fitness level of each subject (VO_2_ max). (Table 2)

### Influence of Adipose Tissue Characteristics on Ethnic Differences in Insulin, Lipids and Adiponectin

**Figure 1:**
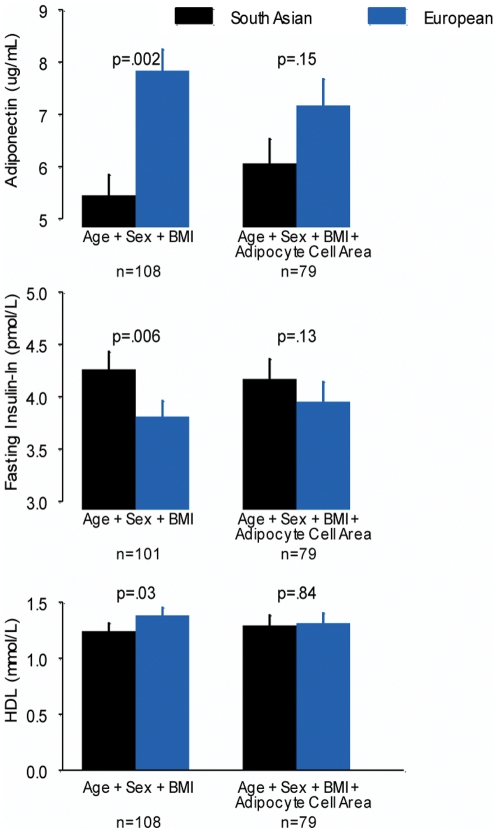
BMI: Body Mass Index, error bars represent the standard error.

Ethnic differences in fasting insulin, HDL cholesterol and adiponectin were not altered after adjustment for body fat distribution (waist to hip ratio, percentage body fat or deep/visceral to superficial fat ratio) but were attenuated after adjustment for adipocyte area. (Figure 1)

### Liver Fat Infiltration

**Figure 2:**
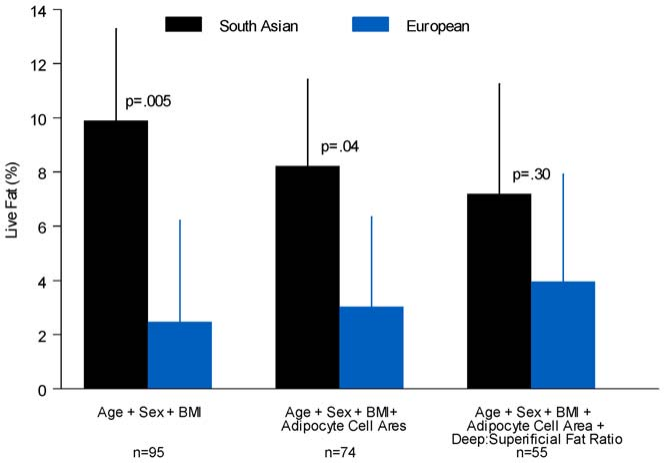
BMI: Body Mass Index, error bars represent the standard error.

The presence of fat infiltration in the liver was associated with significant changes in metabolic risk factors. (Table 3) Individuals classified in the highest tertile of liver fat infiltration were older, more likely to be male, South Asian, and to have a higher BMI (P = 0.0002). Fasting insulin (P = 0.009), fasting triglycerides (P = 0.03), and adipocyte size (P = 0.02), remained significantly associated with increasing liver fat infiltration after adjustment for age, sex, and BMI. (Table 3) To test the adipose tissue overflow hypothesis [6], we adjusted the ethnic difference in liver fat infiltration for differences in adipocyte area and additionally for the relative amount of abdominal deep/visceral to superficial subcutaneous fat. Adjustment for these factors attenuated the ethnic difference in liver fat infiltration (7.2% +/−2.0 vs 4.0% +/−2.0; P = 0.30). (Figure 2)

**Table 3:** Risk factor profile by liver fat tertiles.

	Liver fat tertile group: Mean (SE)				
	Tertile 1	Tertile 2	Tertile 3	P-value*	P-value**
**Number/Group**	31	32	32		
**South Asian: N (%)**	11 (35.5)	19 (59.4)	22 (68.8)		
**Age (years)**	31.77 (1.81)	36.94 (1.66)	40.34 (1.30)		
**Body Mass Index (kg/m^2^)**	24.60 (0.72)	27.07 (0.86)	30.59 (0.87)	0.0002	
**Waist to Hip Ratio**	0.82 (0.01)	0.87 (0.01)	0.93 (0.01)	0.002	0.06
**Body Fat (%)**	30.19 (2.18)	34.32 (1.90)	36.60 (1.61)	0.0002	0.16
**Visceral Fat area (cm^2^)**	66.57 (5.46)	124.94 (13.09)	169.15 (14.48)	0.0018	0.37
**Subcutaneous Fat area (cm^2^)**	205.51 (21.87)	247.89 (19.24)	319.68 (22.87)	0.001	0.63
**Adipocyte Area (x100 units^2^)**	365.85 (16.30)	419.53 (18.98)	480.78 (18.01)	0.0009	0.02
**Adipocyte Diameter (units)**	231.68 (5.58)	248.08 (6.46)	268.18 (5.32)	0.002	0.04
**Fasting Glucose (mmol/L)**	4.72 (0.07)	5.07 (0.10)	5.27 (0.09)	0.02	0.10
**Fasting Insulin - LN (pmol/L)**	3.55 (0.11)	4.17 (0.15)	4.48 (0.10)	0.0001	0.009
**Fasting Triglycerides (mmol/L)**	0.90 (0.08)	1.37 (0.17)	2.03 (0.22)	0.002	0.03
**Fasting HDL (mmol/L)**	1.41 (0.05)	1.36 (0.06)	1.15 (0.06)	0.03	0.22
**hs-CRP (mg/L)**	1.51 (0.31)	1.73 (0.28)	3.30 (0.93)	0.02	0.65
**Diastolic BP (mm/Hg)**	71.42 (1.41)	73.25 (1.30)	79.28 (1.52)	0.06	0.21
**Adiponectin (ug/mL)**	8.42 (0.70)	6.06 (0.60)	4.63 (0.55)	0.03	0.08

* Adjusted for age and sex.

** Adjusted for age, sex and BMI.

## Discussion

**Figure 3:**
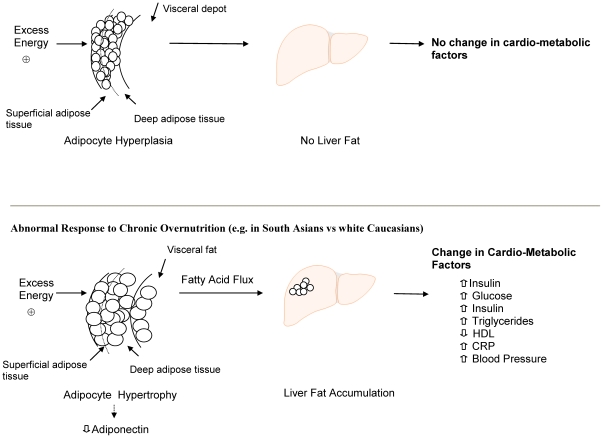
HDL: High Density Lipoprotein, CRP: C-reactive protein.

We observed differences in cardiovascular risk factors and ectopic fat deposition in South Asians compared to white Caucasians which can be explained by increased adipocyte size. This supports the hypothesis that South Asians have a lower capacity to store fat in subcutaneous adipocytes than white Caucasians. Excess fat, therefore, “overflows” to ectopic compartments, in the abdomen and liver [6]. (Figure 3)

Adipose tissue mass expansion occurs by the expansion of pre-existing adipocytes (adipocyte hypertrophy) or by generating new small adipocytes (hyperplasia), or both [17]. Adipocyte hypertrophy usually develops secondary to chronic overfeeding and is associated with metabolic abnormalities including reduced adiponectin, lipolysis from fat cells and increased insulin production [18]–[22]. Altered adipocyte function and storage is also seen in people with congenital and acquired lipodystrophy, as the adipocytes are unable to store triglycerides and high concentrations of fatty acids are shunted to the liver resulting in insulin resistance and dyslipidemia [23]. Our observations are consistent with an “ethnic lipodystrophy” as South Asians have a preponderance of adipocyte hypertrophy, lower adiponectin, reduced superficial subcutaneous fat, increased secondary storage in deep and visceral fat, and higher levels of ectopic fat deposition in the liver compared to people of European origin. This excess delivery of fatty acids to the liver causes fat deposition in the hepatocytes which results in increased hepatic gluconeogenesis, hepatic insulin resistance, and increased production of triglycerides which enter the systemic circulation [24]. Our results are consistent with prior observations in which South Asians compared to white Caucasians were observed to have an excess of fatty liver infiltration and insulin resistance [25]. However our results are only partially congruent with others findings that despite South Asians having an increased adipocyte size in the superficial subcutaneous adipose depot and lower adiponectin, they had *greater* subcutaneous abdominal fat mass and *lower* visceral fat mass as measured by MRI [26]. This difference may be attributed to the differences in study subjects as only young men with a relatively low average BMI of 24 were included. In fact when we restrict our data to match these criteria (South Asian males BMI ≤24) we also observe that South Asians have less visceral fat area compared to their white Caucasian counterparts. Thus, understanding the differences in fat distribution between South Asians and white Caucasians may require inclusion of men and women across wide ranges of BMI as distributional differences may only become apparent when studying overweight and obese individuals.

South Asians have lower lean body mass compared to white Caucasians. We did not observe ethnic variations in IMCL content. IMCL can be influenced by peak exercise capacity [27] and therefore we carefully assessed differences in maximal aerobic capacity and muscle strength and it was not associated with adipocyte size, visceral fat area, adiponectin level, liver fat infiltration, or insulin resistance. These results differ from the observations of two smaller studies conducted in young South Asian men. The first reported that young South Asian men have increased deposition of IMCL compared to Europeans, although no association with IMCL and insulin sensitivity was observed [28]. The second reported an increased IMCL among South Asian men compared to white Caucasian men [25]. Our observations may differ from these studies because we included a larger number of subjects across a wider range of BMI, and because half of our subjects are women. Furthermore, neither of the two previous studies adjusted the IMCL for aerobic capacity or muscle strength. Our findings are consistent with prior observations among young normoglycemic PIMA Indians and other ethnic groups [29] and strongly suggest that IMCL does not account for the marked ethnic difference in metabolic abnormalities we observed between South Asians and white Caucasians.

### Metabolic Markers

We observed that young healthy South Asians have significantly lower levels of adiponectin compared to their white Caucasian counterparts. Low levels of adiponectin are associated with reduced insulin sensitivity, and previous South Asian-white Caucasian comparisons have reported that South Asians have a reduced concentration of adiponectin [30]. On the other hand white Caucasians appear to be relatively protected from reduced adiponectin until they reach very high BMIs i.e. ≥35 [31]. Adiponectin is produced from subcutaneous and visceral adipocytes and the exact proportion of circulating adiponectin attributed to each depot is debated [32], [33]. There is recent evidence demonstrating that adipocyte dysfunction is caused by cellular hypoxia of adipocytes secondary to adipocyte hypertrophy which results in lowered adiponectin synthesis [34]. Large adipocytes are characterized by a reduced production and secretion of adiponectin compared with small newly differentiated adipocytes. In our study the “ethnic difference” in adiponectin was attenuated after adjustment for adipocyte area, which suggests that adipocyte hypertrophy has a significant influence on this biomarker. Thus, our data which draws a strong association between subcutaneous adipocyte hypertrophy and low adiponectin suggest that adipocyte hypertrophy is an important pathophysiologic step in the overflow of fatty acids from subcutaneous fat to deep visceral fat, to liver fat which is marked by lowered adiponectin. The clinical relevance of our findings are therefore, that the presence of abdominal obesity and/or cardio-metabolic risk factors including elevated insulin, low HDL, or low adiponectin in South Asian patients should prompt clinicians to assess the patient for the presence of fatty liver, and prescribe health behaviour changes and/or medical therapy to try and normalize their risk factor profile.

There are a number of implications of our study. First, young, apparently healthy South Asians have greater metabolic impairment compared to white Caucasians who tend to develop metabolic changes at higher levels of obesity and at a more advanced age. Our data helps to explain why South Asians suffer an excess burden of type 2 diabetes and coronary heart disease compared with white Caucasians [35], and supports earlier screening for abdominal adiposity and elevated glucose among South Asians. Second, the metabolic changes observed in South Asians may be prevented by avoiding chronic over nutrition, thereby preventing its consequences (including adipocyte hypertrophy, abdominal adiposity, and ectopic fat deposition). Third, adipocyte cell area and metabolic risk factors may be reduced by reduction of daily energy intake, increased energy expenditure, or by shifting fat deposition from ectopic sites to subcutaneous depots using pharmacologic agents (i.e. PPAR gamma agonists). The limitations of our study include its cross-sectional design, which precludes determination of the temporal nature of the associations between chronic over nutrition, adipocyte hypertrophy, ectopic fat deposition, and metabolic changes, and its relatively small size. However, we assembled a relatively healthy population across a wide range of BMI excluding those with established CVD or type 2 diabetes in order to minimize the effect of medications, dietary changes, or advanced disease pathophysiology. Furthermore, we observed some intriguing ethnic differences by sex, most notably South Asian women's propensity toward liver fat infiltration compared to white Caucasian women. However, the small sample size precluded any detailed study of interactions by sex. Ideally our findings should be reproduced in larger prospective studies to assess if adipocyte hypertrophy is a significant predictor of the incident development of fatty liver infiltration and type 2 diabetes, as well as in randomized trials in which interventions which reduce adipocyte hypertrophy or ecoptic fat deposition are tested. Finally, we did not collect information on whether South Asians were first or second generation immigrants to Canada. This may have introduced heterogeneity in our findings as the time since immigration to Canada influences diabetes risk [36]. However, most studies of South Asian immigrants have reported that the propensity to develop insulin resistance and greater adiposity exists regardless of the first or second generation immigrant status [37].

### Summary

South Asians have an increased adipocyte area compared to white Caucasians. This difference accounts for the ethnic differences in insulin, HDL cholesterol, adiponectin, and the ectopic fat deposition in the liver. Interventions which reduce adipocyte area may improve the metabolic profile of South Asians.
